# Pharmacogenetics of FAC chemotherapy side effects in breast cancer patients

**DOI:** 10.1186/1897-4287-13-S2-A10

**Published:** 2015-11-26

**Authors:** Karolina Tecza, Jolanta Pamula-Pilat, Joanna Lanuszewska, Ewa Grzybowska

**Affiliations:** 1 Center for Translational Research and Molecular Biology of Cancer, Maria Sklodowska-Curie Memorial Cancer Center and Institute of Oncology, Gliwice, Poland

## 

Apart from drug resistance, the patients' oversensitivity to chemotherapy is one of the most serious problems in cancer treatment. Cytotoxic drugs aim the intensively proliferating cancer cells, but unfortunately these drugs also destroy other cells and tissues with high proliferation rates, such as epithelia in gastro-intestinal track and cells in the bone marrow and skin, leading to chemotherapy-related toxicities. Furthermore, these adverse reactions are also observed in relatively slow proliferating tissues in organs involved in systemic detoxification and clearance, i.e. in liver and kidneys.

The most common breast cancer chemotherapy regime is FAC, which combines 5-fluorouracil, doxorubicin and cyclophosphamide. These drugs on the cellular level are responsible for genetic material damage leading to cell cycle checkpoints activation and cell death. Because of the complexity of cellular pathways of each FAC drug, it is expected that molecular mechanisms causing development of adverse reactions to these three drugs are very complex.

Relationships between genetic polymorphisms and chemotherapy-induced toxicity were analyzed in group of 200 breast cancer patients treated with FAC regime in first-line chemotherapy. 13 genetic modifications were selected for this study, including functional variants in genes encoding proteins involved in FAC drugs transport (*ABCB1, ABCC2, ABCG2*), metabolism (*CYP2C19, GSTT1, GSTM1, GSTP1, TYMS, MTHFR*) and drug-induced damage repair (*ERCC1, ERCC2, XRCC1*).

Multifactorial pharmacogenetic models were possible to establish for treatment-related overall and early anemia (low hemoglobin as well as anisocytosis), hepatotoxicity and gastro-intestinal symptoms (nausea). Genetic variants responsible for high risk of these toxicities belonged to transport and metabolic pathways of all three FAC drugs, which confirm the complex causative network of factors underlining systemic adverse reaction to treatment. Furthermore, accumulation of the unfavorable genotypes was responsible for drastic increase of toxicity risk- OR 8.29 and 31.5 for, respectively, overall and early anemia, OR 4.36 and 8.36 for overall and early anisocytosis, OR 7.63 for overall hepatotoxicity and OR 3.07 for early nausea. Potentially life-threatening severe neutropenia correlated with only one genetic polymorphism, but nonetheless we observed over twofold increase in toxicity risk for common allele of VNTR variant in *TYMS *gene.

The results suggest that accumulated modifications in transport, metabolic and damage repair FAC drugs pathways are strongly responsible for systemic unfavorable reaction to treatment. It is believed, that comprehensive model consisting of many genetic alterations could make a promising potential predictive tool of treatment-related toxicity for the 5-fluorouracil, doxorubicin and cyclophosphamide-treated breast cancer patients.

## Acknowledgements

This work was financially supported by National Science Centre grant no. 2012/07/N/NZ5/00026.

